# Calculating percent depth dose with the electron pencil‐beam redefinition algorithm

**DOI:** 10.1120/jacmp.v8i2.2443

**Published:** 2007-04-19

**Authors:** Michael J. Price, Kenneth R. Hogstrom, John A. Antolak, R. Allen White, Charles D. Bloch, Robert A Boyd

**Affiliations:** ^1^ Department of Radiation Physics The University of Texas MD Anderson Cancer Center Houston Texas U.S.A.; ^2^ The University of Texas Graduate School of Biomedical Sciences at Houston Houston Texas U.S.A.; ^3^ Department of Physics and Astronomy Louisiana State University Baton Rouge Louisiana U.S.A.; ^4^ Radiation Oncology Mayo Clinic Rochester Minnesota U.S.A.; ^5^ Department of Biomathematics The University of Texas MD Anderson Cancer Center Houston Texas U.S.A.; ^6^ Mary Bird Perkins Cancer Center Baton Rouge Louisiana U.S.A.

**Keywords:** electron dose algorithm, pencil beam, depth dose

## Abstract

In the present work, we investigated the accuracy of the electron pencil‐beam redefinition algorithm (PBRA) in calculating central‐axis percent depth dose in water for rectangular fields. The PBRA energy correction factor *C*(*E*) was determined so that PBRA‐calculated percent depth dose best matched the percent depth dose measured in water. The hypothesis tested was that a method can be implemented into the PBRA that will enable the algorithm to calculate central‐axis percent depth dose in water at a 100‐cm source‐to‐surface distance (SSD) with an accuracy of 2% or 1‐mm distance to agreement for rectangular field sizes ≥2×2 cm. Preliminary investigations showed that *C*(*E*), determined using a single percent depth dose for a large field (that is, having side‐scatter equilibrium), was insufficient for the PBRA to accurately calculate percent depth dose for all square fields ≥2×2 cm. Therefore, two alternative methods for determining *C*(*E*) were investigated. In Method 1, *C*(*E*), modeled as a polynomial in energy, was determined by fitting the PBRA calculations to individual rectangular‐field percent depth doses. In Method 2, *C*(*E*) for square fields, described by a polynomial in both energy and side of square *W* [that is, C=C(E,W)], was determined by fitting the PBRA calculations to measured percent depth dose for a small number of square fields. Using the function *C*(*E, W*), *C*(*E*) for other square fields was determined, and *C*(*E*) for rectangular field sizes was determined using the geometric mean of *C*(*E*) for the two measured square fields of the dimension of the rectangle (square root method). Using both methods, PBRA calculations were evaluated by comparison with measured square‐field and derived rectangular‐field percent depth doses at 100‐cm SSD for the Siemens Primus radiotherapy accelerator equipped with a 25×25‐cm applicator at 10 MeV and 15 MeV. To improve the fit of *C*(*E*) and *C*(*E, W*) to the electron component of percent depth dose, it was necessary to modify the PBRA's photon depth dose model to include dose buildup. Results showed that, using both methods, the PBRA was able to predict percent depth dose within criteria for all square and rectangular fields. Results showed that second‐ or third‐order polynomials in energy (Methods 1 and 2) and in field size (Method 2) were typically required. Although the time for dose calculation using Method 1 is approximately twice that using Method 2, we recommend that Method 1 be used for clinical implementation of the PBRA because it is more accurate (most measured depth doses predicted within approximately 1%) and simpler to implement.

PACS number: 87.53.Fs

## I. INTRODUCTION

One of the practical goals for an electron‐beam dose algorithm is that the algorithm be able to calculate dose delivered to the patient with an accuracy of 4% in low dose gradient regions or 2 mm distance to agreement (DTA) in high dose gradient regions.^(1)^ Boyd et al.^(2)^ showed that, with the inclusion of an incident energy spectrum, the pencil‐beam redefinition algorithm (PBRA) of Shiu and Hogstrom^(3)^ could meet this standard in water at a 100‐cm source‐to‐surface distance (SSD) on a Varian Clinac 2100C (Varian Medical Systems, Palo Alto, CA). Boyd et al.^(4)^ also showed that the addition of a dual‐source model to account for collimation‐scattered electrons allowed the PBRA to meet this standard in water at 100‐cm and 110‐cm SSDs for selected energy‐applicator combinations on a Varian Clinac 1800. Furthermore, Boyd et al.^(5)^ and Boyd^(6)^ showed that, in heterogeneous phantoms and in patients, dose differences exceeded these criteria only over very small volumes. However, radiation oncologists at MD Anderson Cancer Center believe that the dose inaccuracies encountered should not affect treatment‐planning decisions or patient outcomes.

To date, all investigations into the accuracy of the PBRA have been performed using open‐applicator electron beams. Before any electron‐dose algorithm—in this case the PBRA—can be judged acceptable for clinical implementation, the accuracy of calculated dose to water for all applicator, insert, and SSD combinations should be demonstrated for each beam energy commissioned.

The PBRA expresses calculated dose as a percentage of the central‐axis dose maximum in water for the beam of interest (specified by energy, SSD, and reference rectangular field size). The PBRA dose is calculated assuming perfect collimation—that is, all electrons stop in the insert (no scatter off the aperture edges), and the bremsstrahlung dose is not altered. In the patient, the PBRA does not model large‐angle scattering of primary electrons nor large energy‐loss processes (delta‐ray production and bremsstrahlung). The PBRA compensates for these and other processes that are not modeled first by fitting the incident energy distribution to the distal falloff of the percent depth dose for the open‐applicator or large‐field (one having side‐scatter equilibrium) depth dose.^(2)^ Then, the energy‐dependent correction factor *C*(*E*) is used to force the PBRA‐calculated central‐axis percent depth dose to closely match the percent depth dose measured in water.

**Figure 1:**
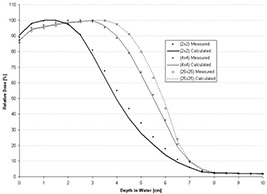
Comparison of percent depth doses measured and calculated by pencil‐beam redefinition algorithm (PBRA) in water for a 15‐MeV beam, 25×25‐cm applicator, for various field‐size inserts in the 25×25‐cm applicator. All PBRA calculations used the energy correction factor *C*(*E*) for the 25×25‐cm field.

**Figure 2:**
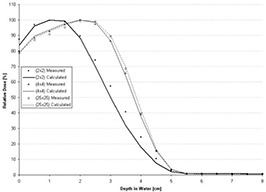
Comparison of percent depth doses measured and calculated by pencil‐beam redefinition algorithm (PBRA) in water for a 10‐MeV beam, 25×25‐cm applicator. All PBRA calculations used the energy correction factor *C*(*E*) for the 25×25‐cm field.

However, using the *C*(*E*) determined for the central‐axis percent depth dose of the open applicator, the foregoing formalism is insufficient for the PBRA to accurately predict central‐axis percent depth dose in water for insert‐shaped fields. Figs. 1 and 2 show comparisons of PBRA‐calculated depth doses and measured depth doses for 2×2‐cm, 4×4‐cm, and 25×25‐cm square fields for the 25×25‐cm applicator‐equipped Siemens Primus (Siemens Medical Solutions U.S.A., Malvern, PA) 15‐MeV and 10‐MeV electron beams respectively. The measured percent depth doses for the open 25×25‐cm and 4×4‐cm fields are predicted within 2% at both energies. However, the 2×2‐cm measured percent depth dose is under‐predicted by PBRA calculations for the 15‐MeV beam in the falloff region by an average of 3% between the depths of 2 cm and 7.5 cm, with the greatest difference being 6% (or 3.5 mm) at a depth of 5 cm. In a similar fashion, the measured 2×2‐cm percent depth dose for the 10‐MeV beam is systematically under‐predicted by the PBRA at all depths less than 5 cm, with the greatest difference of 9% (or 2.5 mm) at a depth of 3.5 cm. These differences exceed the 4% dose and 2‐mm DTA criteria, independent of errors resulting from patient heterogeneity or other factors.

This problem is analogous to that of the conventional pencil‐beam algorithm (PBA) of Hogstrom et al.^(7)^—that is, using only a single, large‐field depth dose, neither the PBA nor the PBRA can predict dose with sufficient accuracy for small‐field depth dose. The PBA incorporated a formalism whereby rectangular‐field percent depth dose was required as input into the algorithm.^(^ ^7^ ^,^ ^8^ ^)^ Rectangular‐field percent depth dose was determined using the square root method from depth doses for two square fields, which were interpolated from a family of measured percent depth dose curves.

Thus, the goals of the present study were to implement methods into the PBRA that would enable the algorithm to calculate central‐axis percent depth dose in water at a 100‐cm SSD and to test whether each method has an accuracy of 2% dose or 1‐mm DTA for rectangular field sizes of 2×2 cm or greater. The two methods investigated modified the manner in which the PBRA calculates and utilizes *C*(*E*). Method 1 determines the *C*(*E*) that best fits the PBRA‐calculated percent depth dose to the measured one for a specific rectangular field size. In other words, *C*(*E*) is determined for each individual field, requiring that “measured” percent depth dose data be utilized, analogous to the PBA. Method 2 determines *C*(*E, W*) as a function of both energy and side of square‐field size *W*, by fitting a set of PBRA‐calculated percent depth doses to a small but comprehensive set of measured square‐field percent depth dose curves. Subsequently, the PBRA dose, calculated as percent of the central‐axis dose maximum in water, can be converted to absolute dose [cGy per monitor unit (MU)] by multiplying by dose output in water, which is energy‐, SSD‐, applicator‐, and rectangular field insert (>2×2 cm)‐dependent and can be stored in a lookup table.

## II. METHODS AND MATERIALS

### A. PBRA methodology

Shiu^(9)^ originally presented the formalization of the PBRA on which the present work is based.^(3)^ Dose delivered to a water mini‐phantom in the patient by an electron beam is divided into two components, that is,

(1) $$D(x,y,z)=D_{e^{-}}(x,y,z)+D_{\gamma }(x,y,z)\;\;,$$

where $D_{e^{-}}$ is the electron dose component, $D_{\gamma }$ is the photon dose component, and *D*(*x,y,z*) is the dose calculated at point (*x,y,z*) in the patient. The calculated electron dose component is determined by summing the dose components of finite bin width (*ΔE*) of the energy distribution, that is,

(2) $$D_{e^{-}}^{calc}(x,y,z)=\beta_{f}\sum_{m=1}^{NE}\phi_{m}(x,y,z)\cdot \frac{S}{\rho }({\bar{E}}_{m}(x,y,z))\cdot C({\bar{E}}_{m}(x,y,z))\;\;,$$

where $\varphi_{m}(x,y,z)$ is the electron fluence of the *m*th energy bin at the point (*x,y,z*), $\frac{S}{\rho }({\bar{E}}_{m})$ is the mass collisional stopping power of water evaluated at the mean energy ${\bar{E}}_{m}(x,y,z)$ of the electrons in the *m*th energy bin at (*x,y,z*), *NE* is the total number of energy bins, and $C({\bar{E}}_{m})$ is the energy‐dependent correction factor. The normalization factor $\beta_{f}$ is a function of field size and is incorporated only for convenience [that is, so that the PBRA generates values of *C*(*E*) near unity]. To do so, the maximum of the uncorrected [C(E)=1], PBRA‐calculated, central‐axis depth dose is normalized to 100%, that is,

(3) $$\beta_{f}=\frac{100}{{\left[\sum_{m=1}^{NE}\phi_{m}(0,0,z)\cdot \frac{S}{\rho }({\bar{E}}_{m}(0,0,z))\right]}_{\max\;\;\;\;\;\;\cdot }}$$

The *C*(*E*) is determined by fitting the PBRA‐calculated electron component of central‐axis dose $D_{e^{-}}^{calc}(0,0,z)$ given by equation 2, to the electron component of the measured dose $D_{e^{-}}^{meas}(0,0,z)$ determined using equation 1—that is, subtracting the photon‐dose component from the measured central‐axis percent depth dose.

Heretofore, the PBRA model did not model electron buildup of the photon‐dose component within the first few millimeters of medium. That approach incorrectly placed the corresponding electron buildup in the electron‐dose component, which resulted in values of *C*(*E*) that were unrealistic, because *C*(*E*) tries to correct for any physics not modeled. A similar issue occurred for electron buildup from electron‐electron scatter (delta‐ray production). It was determined that the buildup of secondary electrons resulting from brems Strahlung radiation and delta‐ray production cannot be ignored; therefore, electron buildup was incorporated into the PBRA photon model by

(4) $$D_{\gamma }(z)=\{\begin{array}{ll} D^{meas}(R_{p}+1)\cdot {\left(\frac{SAD+R_{p}+1}{SAD+z}\right)}^{2}-\delta , & z=z_{1}\\ D^{meas}(R_{p}+1)\cdot {\left(\frac{SAD+R_{p}+1}{SAD+z}\right)}^{2}, & z_{2}\leq z\leq R_{p}+1,\\ D^{meas}(z), & z\geq R_{p+1.} \end{array}$$

where δ, the rapid buildup in dose, is equal to

(5) $$\delta =\left(D^{meas}(z_{2})-D^{meas}(z_{1})\right)+\left(D^{meas}(z_{2})-D^{meas}(z_{3})\right)\;,$$

where $D_{\gamma }(z)$ is the photon‐dose component at depth *z*, $D^{\text{meas}}(z)$ is the measured percent depth dose at depth *z*, $R_{p}$ is the practical range in centimeters,^(10)^ and the source‐to‐axial distance (SAD) is set to 100 cm. In the present study, $z_{1}$ is 0.0 cm, $z_{2}$ is 0.5 cm, and $z_{3}$ is 1.0 cm.

### B. PBRA beam commissioning

Commissioning of the PBRA beam requires determination of several parameters and their validation. These parameters include

- the initial overall angular spread about the mean projected electron direction, $\sigma_{\theta BB}$.
- the drift space, $L_{0}$, which is the effective distance between isocenter and the final plane of the collimator (where the planar source is defined).
- ${\text{SAD}}_{\text{vir}}$, the virtual source distance from isocenter.
- the energy spectrum of the incident electron beam.

The first three parameters are identical to those of the PBA and are determined using conventional methods.^(^ ^7^ ^,^ ^8^ ^,^ ^11^ ^)^ The initial energy spectrum is unique to the PBRA and was calculated using the methods of Boyd et al.^(2)^

To validate that the initial energy spectra were appropriate, uncorrected [C(E)=1] PBRA‐calculated percent depth dose data were compared to measured percent depth dose data. Measured and calculated depth dose curves were both normalized to 100% at dose maximum. The most probable energies of the nominal 15 MeV and 10 MeV electron initial spectra are 14.2 MeV and 9.6 MeV respectively. As previously reported by Boyd et al.,^(2)^ the proper energy spectrum provided good agreement between PBRA‐calculated and measured data on the descending portion of the depth dose curve.^(12)^

The geometric parameters ${(\text{SAD}}_{\text{vir}},L_{0},\;and\;\sigma_{\theta BB})$ were validated by comparisons of measured and PBRA‐calculated off‐axis ratio (OAR) profiles at 100‐cm SSD (isocenter) and at an extended SSD of 110 cm.^(12)^ Off‐axis beam profiles were measured at 1‐cm and 2‐cm depths for energies less than 10 MeV and greater than 10 MeV respectively. Agreement of the locations of the 50% OARs (that is, full‐width half‐maximum) at both SSDs validated ${\text{SAD}}_{\text{vir}}$. Agreement of penumbra shape (80%−20%) at both SSDs validated $L_{0}$ and $\sigma_{\theta BB}$.

### C. Determining the correction factors *C*(*E*) and *C*(*E, W*)

#### *C.1 Method 1: determining C(E) by fitting rectangular field size—specific percent depth dose*

For Method 1, the energy‐dependent correction factor *C*(*E*) was determined by first fitting the PBRA‐calculated electron percent depth dose to the measured electron percent depth dose according to Boyd et al.^(2)^ To do so, *C*(*E*) was modeled as a polynomial of order ε, linear in parameters $a_{\kappa }$, that is,

(6) $$C(E)=\sum_{\kappa =1}^{ɛ}a_{\kappa }\cdot E^{\kappa -1}\;\;\cdot$$

The parameters $a_{\kappa }$ of equation 6 are determined using a least‐squares fit to the measured percent depth dose^(2)^ with field‐size dimensions of the specified rectangle. The fit minimizes the objective function

(7) $$LS_{\nu }=\sum_{i=1}^{ND}{\left[D_{e^{-}}^{calc}(0,0,z_{i})-D_{e^{-}}^{meas}(0,0,z_{i})\right]}^{2}/\nu \;\;,$$

where $D_{e^{-}}^{meas}$ is the measured electron‐dose component of equation 1 at $\left(x=0,\;y=0,\;z=z_{i}\right)$ for the rectangular field, *ND* is the number of data points in the fit, and v=ND−ɛ is the number of degrees of freedom for the fit. This procedure must be repeated to calculate a unique *C*(*E*) for each field size.

#### C.2 Method 2: determining *C*(*E, W*) by fitting a set of square field size—dependent depth doses

For each beam energy, a set of PBRA‐calculated percent depth dose curves for multiple field sizes was fit to a corresponding set of measured percent depth dose curves. The field sizes ranged from a 2×2‐cm field to a square field of a dimension just large enough for the measured data to have side‐scatter equilibrium (percent depth dose remains constant within 1%). Field‐size dependence is incorporated into the calculation of *C*(*E*)–that is, C=C(E,W), where *W* is the side of a square field in centimeters. This incorporation assumed that the coefficients of the polynomial that previously determined *C*(*E*) as a function of energy had a field‐size dependence, that is,

(8) $$C(E,W)=\sum_{\kappa =1}^{ɛ}\left[\sum_{\lambda =1}^{\zeta }a_{\lambda ,\kappa }W^{\lambda -1}\right]E^{\kappa -1}\;\;,$$

where ε and ζ are the degrees of the polynomials describing, respectively, the energy and field‐size dependence. The fitting coefficients $\left\{a_{\lambda ,\kappa }\right\}$ of the energy and field‐size dependence were indexed by κ and λ, respectively; *W* was the side of the square field (in centimeters); and *E* was the energy (in MeV) for which *C*(*E, W*) was calculated.

The coefficients $\left\{a_{\lambda ,\kappa }\right\}$ were determined by minimizing the least squares:

(9) $$LS_{\nu }=\sum_{f=1}^{NF}\sum_{i=1}^{ND}{\left[D_{e^{-}}^{calc,f}(0,0,z_{i})-D_{e^{-}}^{meas,f}(0,0,z_{i})\right]}^{2}/\nu \;\;,$$

where *NF* is the number of field sizes for which measured, central‐axis percent depth doses are used in the fit; *ND* is the number of depths for which depth dose data are available; $\nu =ND\cdot NF-ɛ\cdot \zeta .D_{e^{-}}^{calc,f}(0,0,z_{i})$, calculated from equation 2, is the PBRA‐calculated electron dose component for the *f*th field size at depth $z_{i}$; and $D_{e^{-}}^{meas,f}(0,0,z_{i})$, calculated from equation 1, is the electron component of the measured percent depth dose for the *f*th field size at depth $z_{i}$. All minimizations were performed using a Gauss–Jordan elimination‐based linear least‐squares fitting, determining optimal values for $a_{\kappa }$ and $a_{\lambda ,\kappa }$.^(13)^

The objective behind this method was that, by fitting the correction factor for a finite set of field sizes, the correction factor for other field sizes can be calculated by the model without the need for further fitting.

This approach raises a question: Which field sizes are to be included in the fitting?

Because percent depth dose varies significantly with field size for the smallest fields, percent depth dose for the smallest field is included, and field sizes are more closely spaced for the smaller field sizes. The largest field size included in the fit is the smallest field size for which side‐scatter equilibrium exists. Because the depth dose remains constant, the inclusion of larger field sizes would compromise all fits because of the polynomial dependence of *C*(*E,W*) on field size. Therefore, *C*(*E,W*) for field sizes greater than the maximum field size used in the fit were not determined using the related field size, but rather the maximum field size used in the fit.

To select the order of field size and energy dependence of the polynomial used to model *C*(*E, W*), both the value of the fit and the number of calculated points adhering to the specified criteria were considered. The value of $LS_{v}$ was calculated for varying combinations of ε and ζ. Because higher‐order polynomials can result in nonrealistic or even negative values for *C*(*E, W*), ε was limited to 5. In Method 2, ζ was limited to the number of field sizes whose depth doses were used in the fit.

From these fits, the set of polynomials having $LS_{v}<1.5$ was selected—a selection that should have resulted in most calculated data points being within 2% of measured values. Within this subset, the polynomials with the least number of calculated depth dose points exceeding criteria were selected. Of these polynomials, the one consisting of the least number of terms was chosen to model *C*(*E, W*).

#### C.3 Determining the correction factors *C*(*E*) and *C*(*E,W*) for rectangular fields

To test each method's ability to predict rectangular‐field percent depth doses, PBRA calculations were compared with rectangular‐field depth doses. Rectangular‐field depth doses were derived by the square‐root method^(^ ^7^ ^,^ ^14^ ^)^ and measured square‐field percent depth dose curves.

For Method 1, each rectangular‐field percent depth dose was calculated using a unique *C*(*E*), which was generated by fitting the calculated to the derived rectangular‐field percent depth dose.

For Method 2, the photon and electron components of the percent depth dose were determined using square‐field results. For the electron percent depth dose component, the PBRA first calculated the uncorrected depth dose of the rectangular field for each energy bin. Using equation 2, the result was corrected using the correction factor generated by applying the square‐root method to the energy‐dependent correction factors of two square fields whose sides correspond to the dimensions of the rectangular‐field depth dose, that is,

(10) $$C(E,LxW)=\sqrt{C(E,LxL)\cdot C(E,WxW)}\;\;,$$

where *L* and *W* are the length and width of the rectangular field. The photon component of the rectangular‐field percent depth dose was interpolated from a set of square‐field photon‐dose components. The side of the equivalent square used for interpolation was determined using the method of Sterling et al.,^(15)^ $L_{eq}=2LW/(L+W)$. For rectangular fields, $\beta_{f}$ is estimated by interpolating a value for the equivalent square field from $\beta_{f}$ values for square fields.

#### D. Measured data set

The measured data set was taken using a Siemens Primus linear accelerator at nominal electron beam energies of 15 MeV and 10 MeV. The set consisted of

- central‐axis percent depth doses measured at 100 cm SSD for multiple square‐field sizes (2×2‐cm to 25×25‐cm), and
- selected off‐axis beam profiles measured at a 2‐cm depth.

The set of measured percent depth doses were used to evaluate the effectiveness of the proposed methodologies for predicting field‐size‐dependent depth dose. Off‐axis beam profiles and selected depth dose data were used to commission the PBRA for use in the present study.

All relative percent depth‐dose and off‐axis profile measurements were performed in accordance with American Association of Physicists in Medicine TG‐25^(10)^ using two silicon‐diode electron field detectors (EFD^3G^: Scanditronix–Wellhöfer, Schwarzenbruck, Germany) in conjunction with a water phantom and scanner (WP‐700: Wellhöfer Dosimetry, Schwarzenbruck, Germany). After initial detector setup, to ensure that the setup and characteristics of the electron beam remained constant, a 10‐MeV 25×25‐cm open‐field depth dose was scanned at the beginning of each measurement session. That scan was also repeated at the end of each measurement session. Differences between session readings at $R_{100}$ and $R_{50}$ that were no greater than 0.3% were deemed acceptable.

Off‐axis beam profiles were taken in a cross‐plane orientation, perpendicular to plane of electron bending. The width of each scan was at least 8 cm wider than the measured field size. For example, a 15×15‐cm off‐axis profile would be measured by scanning a total length of 24 cm. Percent depth dose was measured along the central axis. The depth to which measurements were taken was 3 – 4 cm deeper than the practical range. For all diode measurements, the continuous data collection mode was employed. Data were collected continuously for the entire length of the scan, recording a maximum of 20 data points per second. The detector scanning speed was $1.75\;\text{mm}•s^{-1}$, which implies that the spatial resolution of the data sampling was less than 0.1 mm.

Percent depth‐dose and off‐axis profile data were both smoothed post‐measurement using a 31‐point‐window moving‐average method. In addition, off‐axis profiles were corrected for small asymmetries by translating (<1 mm) the central axis to lie midway between the 50% off‐axis dose points. Percent depth dose curves were then normalized to 100% at dose maximum. Off‐axis profiles were normalized by averaging the data ±1 cm about the central axis and scaling that value to 100%.

Random error was assessed in both the low and high dose gradients of the depth dose curve. The low dose gradient was defined by the measured data values lying between $R_{95}$ and $R_{98}$. The high dose gradient was defined by the data between $R_{55}$ and $R_{45}$. The precision of the smoothed data was estimated by comparing its value with that of the unsmoothed data and assuming a normal distribution of error.^(12)^ Results showed that the precision of the smoothed measured data in the regions of low and high dose gradient is 0.1% or less, which is very good and sufficient for the comparisons in the present study.

## III. RESULTS

### A. Using Method 1 to determine *C*(*E*) at 15 MeV

**Figure 3:**
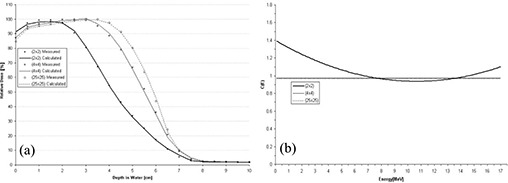
(a) Comparison of beam percent depth doses measured and calculated by pencil‐beam redefinition algorithm (PBRA) in water for a 15‐MeV beam, 25×25‐cm applicator, for various field‐size inserts in the 25×25‐cm applicator. The PBRA calculations used an energy correction factor *C*(*E*) calculated for each field size independently (Method 1, see text). (b) Energy correction factor *C*(*E*) versus energy for PBRA calculations for each field size.

Using Method 1, the polynomial chosen to model *C*(*E*) has no field‐size relationship, because *C*(*E*) is calculated for each field‐size‐dependent depth dose. A comparison of PBRA‐calculated and measured percent depth dose curves is plotted in Fig. 3(a) for the 2×2‐, 4×4‐, and 25×25‐cm field sizes, and the corresponding curves for *C*(*E*) used by the PBRA are plotted in Fig. 3(b). The 25×25‐cm depth dose is fit using a constant *C*(*E*) of 0.97. Only at the surface and in small segments of the falloff region are differences between measured and calculated points greater than 1% found. Therefore, all calculated points are within our accuracy criteria of 2% or 1 mm DTA. The 4×4‐cm depth dose is fit using a constant *C*(*E*) of 0.98. All calculated points are within criteria. In fact, excluding a small segment within the falloff region, they are all within 1% of the measured values. The 2×2‐cm depth dose is fit by modeling *C*(*E*) using a second‐order polynomial. All points are within criteria; however, a systematic under‐prediction (1%−2%) of dose occurs from 0.5 cm to 2.0 cm.

**Figure 4:**
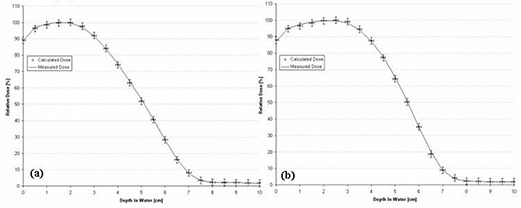
Comparison of percent depth doses measured and calculated by pencil‐beam redefinition algorithm (PBRA) in water for a 15‐MeV beam for (a) a 2×10‐cm field and (b) a 3×12‐cm field. The energy correction factor *C*(*E*) was determined using Method 1 (see text). The error bars correspond to the acceptability criteria (horizontal bars, ±1 mm; vertical bars, ±2%).

A unique *C*(*E*) was calculated for each individual rectangular field size–dependent depth dose. The *C*(*E*) was modeled using a second‐order polynomial for the 2×10‐cm field depth dose and as a constant for the 3×12‐, 3×8‐, and 4×15‐cm fields. Fig. 4 shows the results for the 2×10‐cm and 3×12‐cm fields. All PBRA‐predicted dose points for all fields are within criteria (less than 1% difference between calculated and measured points at all depths).

### B. Using Method 1 to determine *C*(*E*) at 10 MeV

**Figure 5:**
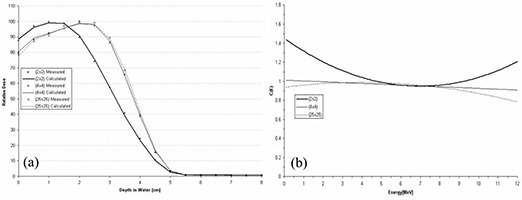
(a) Comparison of beam percent depth doses measured and calculated by pencil‐beam redefinition algorithm (PBRA) in water for a 10‐MeV beam, 25×25‐cm applicator, for various field‐size inserts in the 25×25‐cm applicator. The PBRA calculations used an energy correction factor *C*(*E*) calculated for each field size independently (Method 1, see text). (b) The energy correction factor *C*(*E*) versus energy for PBRA calculations for each field size.

A comparison of PBRA‐calculated and measured percent depth dose curves is plotted in Fig. 5(a) for the 2×2‐, 4×4‐, and 25×25‐cm field sizes, and the corresponding curves for *C*(*E*) used by the PBRA are plotted in Fig. 5(b). The 25×25‐cm depth dose is fit using a *C*(*E*) modeled by a second‐order polynomial in energy. All calculated points are within criteria.

The greatest difference between measured and PBRA‐calculated depth dose occurs at a 2.5‐cm depth in the low dose gradient region (1.2%) and at a 4‐cm depth in the high dose gradient (1.3%). The 4×4‐cm depth dose is fit using a *C*(*E*) modeled by a first‐order polynomial in energy. All PBRA‐calculated depth dose points are within 1% of measured values, excluding $R_{100}$ and a depth of 1.5 cm, where a difference of 1.5% is observed. The 2×2‐cm depth dose is fit using a *C*(*E*) modeled by a second‐order polynomial. All points are within criteria, with 1% underestimation in depth dose at the surface and 1.2% underestimation near the end of the falloff region. In Fig. 5(b), *C*(*E*) is observed to vary the most for the 2×2‐cm field, in this case decreasing rapidly at low energies and increasing rapidly for energies greater than 8 MeV.

**Figure 6:**
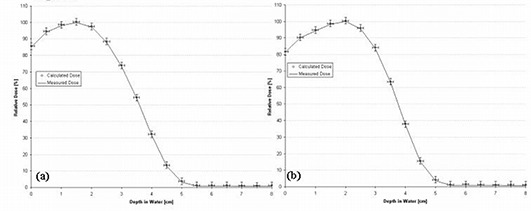
Comparison of percent depth doses measured and calculated by pencil‐beam redefinition algorithm (PBRA) in water for a 10‐MeV beam for (a) a 2×10‐cm field and (b) a 3×12‐cm field. Each energy correction factor *C*(*E*) was determined using Method 1 (see text). The error bars correspond to the acceptability criteria (horizontal bars, ±1 mm; vertical bars, ±2%).

A unique *C*(*E*) was calculated for each individual rectangular field size–dependent depth dose. The *C*(*E*) values associated with 2×10‐cm and 4×15‐cm fields were both modeled by a second‐order polynomial, and the *C*(*E*) for the 3×12‐cm and 3×8‐cm fields were modeled by a first‐order polynomial. Fig. 6 shows the results for the 2×10‐cm and 3×12‐cm fields. All PBRA‐calculated dose points for all fields are within criteria (less than 1% difference between calculated and measured points at all depths).

### C. Using Method 2 to determine *C*(*E, W*) at 15 MeV

**Figure 7:**
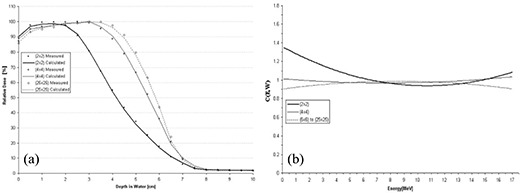
(a) Comparison of percent depth doses measured and calculated by pencil‐beam redefinition algorithm (PBRA) in water for a 15‐MeV beam, 25×25‐cm applicator, for various field‐size inserts in the 25×25‐cm applicator. The PBRA calculations used an energy and side‐of‐square correction factor *C*(*E, W*) fit simultaneously to all fields (Method 2). (b) Comparison of the correction factor *C*(*E, W*) and energy for the PBRA calculations for each field size. Values of *C*(*E, W*) determined using Method 2 were modeled by a second‐order polynomial in field size and energy dependence.

At 15 MeV, the function used to model *C*(*E, W*) was a second‐order polynomial in both energy and field‐size dependence. This was the lowest‐order polynomial determined using Method 2 for which all data points fell within criteria. The reduced least‐squares value equals 1.04. The resulting comparison of PBRA‐calculated with measured percent depth dose curves is plotted in Fig. 7(a) for the 2×2‐, 4×4‐, and 25×25‐cm square fields, and the corresponding curves for *C*(*E, W*) are plotted in Fig. 7(b). The value of *C*(*E, W*) varies most for the smallest field size (particularly at the lower energies), which is similar to the results obtained using Method 1.

Method 2 predicts all points within criteria for the 25×25‐cm depth dose. A slight over‐prediction of depth dose (<0.6%) occurs at the surface and throughout the buildup region, and a small under‐prediction of depth dose (<1.8%) occurs in the falloff region, particularly at depths near $R_{90}$. All 4×4‐cm calculated depth dose points fall within criteria; however, conversely to the 25×25‐cm comparison, calculation under‐predicts depth dose (<0.7%) in the buildup region and over‐predicts depth dose (<1.4%) in the region near $R_{90}$. Although all predicted points are within criteria, a systematic under‐prediction of depth dose (<1.1%) occurs in the buildup region of the 2×2‐cm depth dose curve.

**Figure 8:**
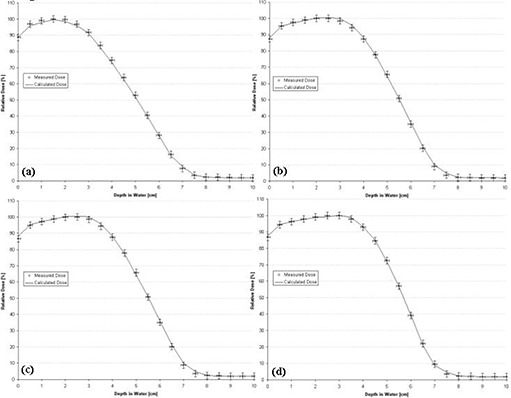
Comparison of percent depth doses measured and calculated by pencil‐beam redefinition algorithm (PBRA) in water for a 15‐MeV beam, for rectangular field sizes of (a) 2×10 cm, (b) 3×12 cm, (c) 3×8 cm, and (d) 4×15 cm. The error bars correspond to the acceptability criteria (horizontal bars, ±1 mm; vertical bars, ±2%).

The 2×10‐, 3×12‐, 3×8‐, and 4×15‐cm rectangular‐field percent depth dose curves calculated by the PBRA are all predicted within criteria by the square‐root method and the nine‐term *C*(*E, W*) polynomial for the square fields. Fig. 8 shows the comparisons for these rectangular‐field percent depth dose curves. In the region near $R_{100}$, PBRA calculations differ from measurements by more than 1% for each rectangular‐field depth dose. A 1.4% under‐prediction occurs at 3.5 cm for the 2×10‐cm field, a 1.5% over‐prediction occurs from 3 cm to 3.5 cm for the 3×12‐cm field, a 1.4% over‐prediction occurs from 3 cm to 3.5 cm for the 3×8‐cm field, and a 1.1% over‐prediction occurs at 3.5 cm for the 4×15‐cm field [compare Fig. 8(a,b,c,d) respectively].

**Figure 9:**
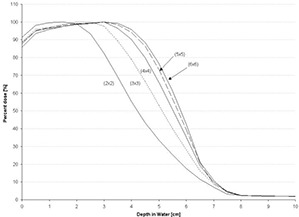
Percent depth doses calculated by pencil‐beam redefinition algorithm (PBRA) at 15 MeV for field sizes 3×3 cm, 5×5 cm, and 6×6 cm—that is, field sizes not used to determine the energy and side‐of‐square correction factor *C*(*E, W*)—are compared to PBRA‐calculated percent depth doses for 2×2‐cm and 4×4‐cm fields, which were fit to the measured data set using Method 2 (see text). Examples are given of the PBRA's ability to predict percent depth doses for field sizes intermediate to those for which a *C*(*E, W*) has been explicitly calculated.

Method 2 differs from Method 1 in that, once *C*(*E, W*) is determined, it is subsequently used by the PBRA in predicting intermediate field sizes. Fig. 9 shows PBRA‐calculated percent depth doses at field sizes intermediate (3×3 cm, 5×5 cm, and 6×6 cm) to those included in the 15 MeV fit (2×2 cm, 4×4 cm, 25×25 cm). These results, which appear quantitatively appropriate, were verified by comparison with measured field sizes, and the two agreed at all depths within our criteria of acceptance.

### D. Using Method 2 to determine *C*(*E, W*) at 10 MeV

**Figure 10:**
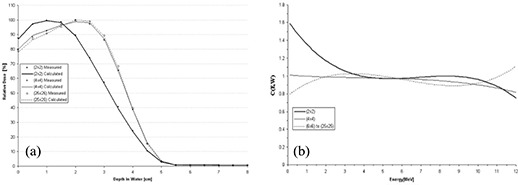
(a) Comparison of percent depth doses measured and calculated by pencil‐beam redefinition algorithm (PBRA) in water for a 10‐MeV beam, 25×25‐cm applicator, for various field‐size inserts in the 25×25‐cm applicator. The PBRA calculations used an energy and side‐of‐square correction factor *C*(*E, W*) fit simultaneously to all fields (Method 2, see text). (b) Energy correction factor *C*(*E, W*) versus energy for PBRA calculations for each field size. Values of *C*(*E, W*) determined using Method 2 (see text) were modeled by a second‐order polynomial in field size and third‐order in energy dependence.

At 10 MeV, the function used to model *C*(*E, W*) was a polynomial second‐order in field‐size dependence and third‐order in energy dependence. This was the lowest‐order fit in which all data points fell within the fitting criteria. The reduced least‐squares value equals 0.63. The resulting comparison of PBRA‐calculated with measured percent depth dose curves is plotted in Fig. 10(a) for the 2×2‐, 4×4‐, and 25×25‐cm fields, and the corresponding curves for *C*(*E, W*) are plotted in Fig. 10(b).

Again, *C*(*E, W*) can be seen to vary most for the smaller field size, particularly at the lower energies. For the 25×25‐cm depth dose, a slight under‐prediction of depth dose (<1%) occurs at the surface and for a small segment of the falloff region (1%). An approximate 1% over‐prediction also occurs at $R_{100}$. Conversely to the 25×25‐cm comparison, the 4×4‐cm calculated depth dose calculation under‐predicts depth dose (1.1%) at $R_{100}$ and slightly over‐predicts depth dose at several depths in the falloff (≈0.9%). Although all predicted points are well within criteria, an under‐prediction of depth dose (<0.8%) occurs in the falloff region and at the surface (0.5%) of the 2×2‐cm depth dose curve.

**Figure 11:**
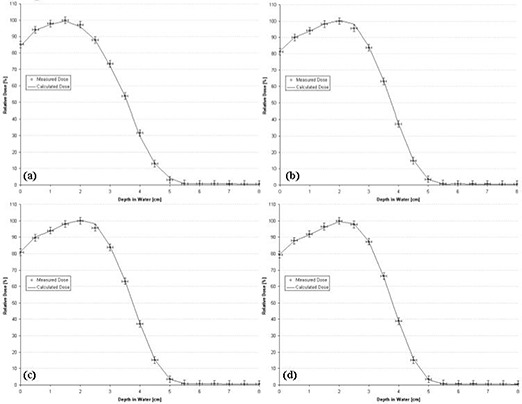
Comparison of percent depth doses measured and calculated by pencil‐beam redefinition algorithm (PBRA) in water for a 10‐MeV beam for rectangular field sizes of (a) 2×10 cm, (b) 3×12 cm, (c) 3×8 cm, and (d) 4×15 cm. The error bars correspond to the acceptability criteria (horizontal bars, ±1 mm; vertical bars, ±2%).

The 2×10‐, 3×12‐, 3×8‐, and 4×15‐cm rectangular‐field percent depth dose curves calculated by the PBRA were all predicted within criteria using the square root method and the 12‐term polynomial for *C*(*E, W*). Fig. 11 shows the comparisons for these rectangular‐field percent depth dose curves. All calculated depth dose points for the four field sizes fall within criteria. The largest errors for the 3×12‐cm and 3×8‐cm depth dose curves occur at a depth of 2.5 cm, where dose is over‐predicted by 2.7% and 2.6% respectively. Because this is a region of high dose gradient and the predicted points are within 1 mm of the measurements, these points fall within criteria.

## IV. CONCLUSIONS

Our results support the hypothesis that a method can be implemented into the PBRA that enables it to calculate central‐axis percent depth dose in water at 100 cm SSD with an accuracy of 2% or 1‐mm DTA. The hypothesis was true for implementing the energy‐dependent correction factor *C*(*E*) using Method 1, and the energy‐dependent and field‐size‐dependent correction factor *C*(*E, W*) using Method 2 for applicator‐equipped Siemens radiotherapy accelerators, for high‐ and low‐energy electron beams, and for rectangular field sizes greater than 2×2 cm.

However, for clinical implementation of the PBRA, we recommend using Method 1 because

- Method 1 can calculate most rectangular‐field depth doses to within approximately 1% of measurement, which is significantly more accurate than results acquired with Method 2, and
- more subjectivity and data analysis are required than are involved in Method 2.

Future development of a fast PBRA algorithm calculating for central‐axis depth dose in water would be useful for Method 1. Although Method 2 is not recommended, it holds promise. Future investigation into the use of a function other than a polynomial to model *C*(*E, W*) and into improved methods for determining *C*(*E, W*) for rectangular fields could make Method 2 more attractive. Both methods could improve with refinement of the selection criteria that determine the order of the polynomial that models the energy correction factor.

## ACKNOWLEDGMENTS

This work was supported in part by a sponsored research agreement with Siemens Medical Solutions U.S.A.

## Supporting information

Supplementary Material
