# Supplementary material for: Calculating percent depth dose with the electron pencil‐beam redefinition algorithm
Source: J Appl Clin Med Phys. 2007 Apr 19;8(2):61–75. doi: 10.1120/jacmp.v8i2.2443 (PMC5722410; doi:10.1120/jacmp.v8i2.2443)
Supplement: Supplementary file 1 — Supplementary Material [file ACM2-8-061-s001.doc]

**Calculating percent depth dose with the electron pencil-beam redefinition algorithm**

**Michael J Price**

*Department of Radiation Physics*

*The University of Texas M D Anderson Cancer Center*

*1515 Holcombe Boulevard, Houston, TX 77070, USA*

[mjprice@mail.mdanderson.org](mailto:mjprice@mail.mdanderson.org)

Calculating percent depth dose with the electron PBRA

**Kenneth R Hogstrom**

*Department of Radiation Physics*

*The University of Texas M D Anderson Cancer Center*

*1515 Holcombe Boulevard, Houston, TX 77070, USA*

*Present address: Department of Physics and Astronomy*

*Louisiana State University*

*202 Nicholson Hall, Tower Drive, Baton Rouge, LA 70803-4001, USA*

**John A Antolak**

*Department of Radiation Physics*

*The University of Texas M D Anderson Cancer Center*

*1515 Holcombe Boulevard, Houston, TX 77070, USA*

*Present address: Department of Radiation Oncology, Desk SR*

*Mayo Clinic*

*200 First St. SW, Rochester, MN 55905, USA*

**R Allen White**

*Department of Biomathematics*

*The University of Texas M D Anderson Cancer Center*

*1515 Holcombe Boulevard, Houston, TX 77070, USA*

**Charles D Bloch**

*Department of Radiation Physics*

*The University of Texas M D Anderson Cancer Center*

*1515 Holcombe Boulevard, Houston, TX 77070, USA*

*Present address: Department of Radiology*

*Baylor College of Medicine*

*One Baylor Plaza, MS 360, Houston, TX 77030, USA*

**Robert A Boyd**

*Department of Radiation Physics*

*The University of Texas M D Anderson Cancer Center*

*1515 Holcombe Boulevard, Houston, TX 77070, USA*

*Present address: Mary Bird Perkins Cancer Center*

*4950 Essen Lane, Baton Rouge, LA, 70809, USA*
